# Correlates of pregnancy among Female Sex Workers (FSWs) in semi urban Blantyre, Malawi

**DOI:** 10.1186/s12884-020-03018-3

**Published:** 2020-06-01

**Authors:** Donatien Twizelimana, Adamson S. Muula

**Affiliations:** 1 Mlambe Mission Hospital, P.O. Box: 45 Lunzu, Blantyre, Malawi; 2 Department of Public Health, School of Public Health and Family Medicine, College of Medicine, University of Malawi, Blantyre, Malawi; 3 The Africa Center of Excellence in Public Health and Herbal Medicine, University of Malawi, Blantyre, Malawi

**Keywords:** Female sex workers, Correlates of pregnancy, Motherhood, HIV, FSWs’ partners

## Abstract

**Background:**

Little is known about female sex workers’ (FSWs) reproductive health apart from their being at higher than usual risk of sexually transmitted infections (STIs), including HIV. The aim of this study was therefore to investigate the correlates of pregnancy among FSWs in semi – urban Blantyre in Malawi.

**Methods:**

We used systematic sampling to recruit a total of 200 FSWs in four different study sites in Blantyre. Data were collected through questionnaire interviews. We calculated the mean and standard deviation for continuous covariates and proportions for categorical variables to describe the data. Logistic regression analysis was used to examine the correlates between the outcome variable (pregnancy) and independent variables.

**Results:**

Ninety one (45, 5%) FSWs were between 18 and 24 years. The prevalence of pregnancy was 61% for FSWs born in rural place as compared to 37% for those who were born in town. In multivariate analysis FSWs who reported to value being respected as mothers had 12 times the risk of pregnancy comparing to the ones who did not (AOR: 11.8, 95% CI: [4.56, 30.72] *p*-value < 0.001). FSWs who reported using condoms inconsistently had five times the risk of pregnancy compared to the ones who did not, (AOR: 5.26, 95% CI: [2.29, 12.08], *p*-value < 0.001). FSWs who had a request to bear children from steady partners had 5 times the risk of pregnancy comparing to the ones who did not (AOR: 5.07, 95% CI: [2.14, 11.99]). FSWs who reported forgetfulness of contraceptives’ use had 3 times more risk of pregnancy comparing to the ones who did not (AOR: 3.49 CI: [1.29, 9.37], *p-*value < 0.013)***.***

**Conclusion:**

The study documents a wide range of correlates of pregnancies among FSWs in the study sites. It is important to recognize the child bearing desires and circumstances of FSWs in order to inform health programs responsive to their needs.

## Background

Little is known about FSWs’ reproductive health apart from their higher than usual risk of sexually transmitted infections, including HIV. Many health promotion programs and researchers on FSWs have focused mainly on HIV infections’ prevention, transmissions, diagnosis and treatment [1, 2]. Although there are some studies on unintended pregnancies among FSWs, there are limited data on the specific contexts FSWs’ experience pregnancies and their intentions to have children [1, 3–8]. The decisions on whether, when and with whom to have children among FSWs in semi – urban Blantyre have not been adequately explored. In attempt to fill this gap, we conducted a cross-sectional quantitative study in semi-urban Blantyre to investigate the correlates of pregnancy among FSWs in the same location.

Other than limited access to reproductive health services due to discrimination, there is also stigma in this marginalized population with consequent high prevalence of HIV, and sexually transmitted diseases due to non-responsive health services [9]. In addition, FSWs operate in a complex environment due to multiple partnership. All these challenges may have an effect on their reproductive health.

Studies have unveiled that there are individual, relational and cultural factors that are associated with FSWs’ desire to have children. Those factors include but are not limited to: bad outcomes with the previous pregnancies, contraceptives unmet needs, steady partners’ request to FSWs to bear children, sexual violence, motherhood’s obligation, and missing contraceptive pills [2, 10]. Study findings also suggest that due to considerable value of children, some FSWs seek pregnancy in sex work, and return to their families as expectant mothers [2, 11]. Pregnancy reflects their achievement and/or their own abilities. After delivery they are satisfied to be called mothers and they are well respected in their communities [2].

About 75% of FSWs in Sub Saharan Africa have at least one child [2, 12]. Estimates of pregnancy among FSWs range from 27% in Madagascar to about 91% in India [2, 13, 14]. In Madagascar a study reported that 250 FSWs out 935 FSWs who were enrolled in a study to promote condom use got pregnant within 18 months of follow up [5]. It can be therefore suggested that not all pregnancies in FSWs community are unintended.

In general the process FSWs get pregnant is the same they become infected or infect their partners with STI including HIV. It implies unprotected heterosexual sex in this community known to have high prevalence of HIV. In the absence or limited prevention of mother to child transmission (PMTCT) services, female sex workers’ unborn babies are also at risk of vertical transmission of HIV [9–13]. Challenges in accessing reproductive health services contributes to unsafe abortion which is prevalent in this community. Study findings suggest that unsafe abortion contributes to high maternal death among this key population. Although the drivers to the intentions to get pregnant are not well known, there are many risks in the paths followed by FSWs to conceive and have children [15]. We believe that those pathways can be improved. We therefore collected quantitative data to evaluate the correlates of pregnancy among FSWs in semi-urban Blantyre, Malawi. The knowledge of those correlates may contribute in designing health promotion intervention to prevent public health problems such as abortion, HIV transmission, unmet needs for contraception. We acknowledge that the unavailability of sufficient information on FSWs reproduction is a big challenge to FSWs fertility programing as well as to the policy makers. We also believe that our study findings are an added knowledge to the current scanty information on FSWs’ reproductive health in semi-urban Blantyre in Malawi. No similar study has ever been done in the same area.

## Methods

The study was conducted between November 2018 and January 2019. In this cross sectional study, we used quantitative methods to collect data on the prevalence and correlates of pregnancies among FSWs, in semi-urban Blantyre. The study was conducted in four townships Chirimba, Lunzu, Kachere, Mbayani located in semi -urban Blantyre, Southern region of Malawi.

FSWs aged between 18 and 49 years, who had exchanged sex for money or goods and who signed the consent were eligible for the study participation. Some FSWs were recruited as seeds in snow ball sampling. They assisted us to have access to FSWs in four purposively selected study sites.

The outcome variable of the study was pregnancy. We had many hypothesized socio-demographic variables and behavioral related characteristics, for instance: missing contraceptive pills, having steady partners, inconsistence condom use, to be respected as a mother, alcohol intake, violence, rape, extension of clans, challenges in accessing contraceptive pills, mothers’ obligation, peer pressure from FSWs, condom breakage or slippage.

### Operational definitions

In this study, *pregnancy* also known as gestation was defined as the maternal condition of having a developing of one or more offspring in the woman’s body. *Female Sex Workers* were defined as women who sell sex in the exchange of money or goods. *Condom breakage* was defined as rupture of a condom during sexual intercourse. *Condom slippage* is getting out of a condom from a penis during sexual intercourse. *Inconsistence condom use* was defined as using a condom sometimes or rarely during sexual intercourse. In heterosexual relationship a steady partner was defined as spouse or co-habiting partner or someone with a romantic relationship with for a long period of time.

The sample size was calculated using single population proportion formula. We used 15% as the prevalence of FSWs trying to conceive with one of their partners [10]. Marginal error of 5%, and at 95 level of confidence. Thus the required sample size was calculated at 196. For convenient purpose we used 200 FSWs as study participants.

At each of the four sites, data collection took place at neutral and confidential places mutually agreed to by the study team and research participants. Fifty FSWs from each site were systematically selected to participate in the study. A specific period was identified within which the questionnaires were completed by the research assistants through the interview process with FSWs.

Female research assistants trained in data collection and research went to each site at a pre-arranged time. They explained the purpose of the study and emphasized the fact that FSWs who do not wish to participate may either leave, or remain in our study but they will not be adversely affected by their voluntary decisions not to participate. Following the explanation, study participants were given an opportunity for questions and clarifications. Each of the FSWs was asked questions by the research assistant and the survey questionnaires were completed by the data collector. Cash reimbursement of Malawi Kwacha (MK) 1500.00 (approximately 2 US$ at the time of data collection) was provided to all study participants as compensation for their time in the study.

For this study we had a database for entry of quantitative data from the completed questionnaires. Data analysis were conducted in Stata 14.1 (Stata Corporation, College Station, TX, USA). Descriptive statistics were used to analyze data from socio-economic and demographic characteristics of the study participants. Logistic regression analysis was used to investigate the relationship between pregnancy (binary outcome) and explanatory variables among FSWs.

### Ethical consideration

The study was approved by COMREC (College of Medicine Research and Ethics Committee), University of Malawi, (certificate number P.07 / 18 / 2444, dated 08 – Sept – 2018). We got clearance from the local authorities before the study started. All study staff were carefully trained in human subjects’ protection, especially the importance of protecting privacy and confidentiality and obtaining informed consent from each study participant using the approved consent forms. Participants were informed of their right to withdraw from the study and not to answer any questions they felt uncomfortable with answering. All the information which was provided by the participants was treated with confidentiality.

## Results

### Socio-demographic characteristics

We recruited a total of 200 study participants (FSWs). Ninety one (45,5%) FSWs were between the age of 18 and 24 years. Ninety eight participants (49%) had steady partners. One hundred and forty six (73%) FSWs interviewed had attended only primary school as their highest level of academic achievement.

More than half were Christian. Nighty eight (49%) of the study participants sell sex in the bars. One hundred and three (67.3%) of the FSWs interviewed grew up in the rural place, and searching for better life was the main reason of leaving the rural place (88% of the respondents). One hundred and twenty six (66.3%) of the respondents reported relying only on the sex work for their income. Business was the main additional work mentioned by the majority of the respondents (80%) (Table 1).

**Table 1:** Socio-demographic and economic characteristics of female sex workers in semi – urban Blantyre 2018 (*n* = 200)

Characteristics	Frequency	Percent (%)
**Age in years (*n* = 200)**		
18–< 24	91	45.5
24–< 29	34	17
29–< 34	32	16
34- < 39	17	8.5
39- < 44	15	7.5
44- < 49	11	5.5
**Relationship status(*n* = 200)**		
Had steady partner	98	49
Had no steady partner	63	31.5
Dissolved (widowed and divorced)	39	19.5
**Educational status (*n* = 200)**		
**Level attained**		
Primary (1–8)	146	73
Secondary (9–12)	47	23.5
Higher education	7	3.5
**Religion *n* = (200)**		
Christianity	133	66.5
Islam	67	33.5
**Place of work** (***n*** **= 186)**		
Night club	98	52.7
Bar	61	32.8
Homes (in-house)	16	8.6
Hotel	11	5.9
**Additional work (*n*** **= 190)**		
Yes	64	33.7
No	126	66.3
**Type of additional work (*n*** **= 60)**		
Business	48	80
Waiter	7	11.7
Housemaid	5	8.3
**Monthly income in USD (*n* = 60)**		
< =50	60	100
**Birth place (*n*** **= 153)**		
Rural	103	67.3
Urban	50	32.7
**Arrived from out of the study site (*n*** **= 200)**		
No	109	54.5
Yes	91	45.5
**Reason of leaving birth place (*n*** **= 91)**		
Searching for better life	80	88
Family collapse	10	11
Fear of early marriage or instability	01	1.0

There was collinearity between mother obligation, to be respected as mothers and peer pressure from fellow female sex workers. There was also collinearity between condom rapture and forgetfulness of contraceptives use (missing contraceptive pills). Challenge in accessing contraceptives was not significant in adjusted crude ratios. We did not find an association between pregnancy and duration in the sex work, mothers’ obligation, rape, alcohol influence, contraceptives’ failure, loss of children and pregnancy, God’s orders fulfillments, and extension of the clan.

## Discussion

To the best of our knowledge this is the first study which investigated the correlates of pregnancy among FSWs in semi urban Blantyre, Malawi. The prevalence of pregnancy was 61% for FSWs born in rural place as compared to 37% for those who were born in town. There is a significant difference between the prevalence of FSWs who were born in rural and urban areas, 103 FSWs as compared to 50 FSWs respectively. According to our study findings 88% of the participants migrated from rural to urban areas searching for better life (Table 1). In general young woman migrate from rural to urban in search for opportunities, especially employment or education. Most of them opt to sex trade in response to economic needs in circumstances where they have few or no other reliable options.

Our study findings suggest that inconsistent condom use with the clients, desire to be respected as mothers, request from a steady partner, and missing contraceptive pills are associated with pregnancy in FSWs community in semi urban Blantyre (Table 2). The strong association between inconsistency condom use and pregnancy among FSWs in our study is consistent with other studies’ findings in other countries [10]. FSWs face multiple barriers to consistent condom use, including limited negotiation power with paying and nonpaying partners, voluntary and/or accidentally condom’s rupture [16], refusal to use condoms because of sexual dissatisfaction [17–19]. Alcohol intake, violence, have been also reported to correlate with inconsistent condom use. Studies done elsewhere also suggest that the more the clients pay for sex the more power they have to dictate unprotected sex [20]. Most FSWs live in poverty and have many responsibilities, such as caring for children and extended families. These challenges make them likely to accept increased pay for unprotected sex which may results in getting pregnant, HIV, or both [20–22]. It is also possible that FSWs who don’t compromise on condomless sex, theirs clients may opt to rupture condoms deliberately without the FSW’s prior knowledge [16]. Sometimes FSWs are put in positions which facilitate condom rupture with the aim to have condomless sex [16, 23]. Study findings from South Africa on action taken after condom breakage or slippage, 36% of the FSWs reported that they continued sexual act up to the end despite having knowledge of the condom failure. After 24 h 53%, admitted that no health care action was taken or sought despite knowing that they can get pregnant or be infected with HIV [23]. Managements for sexual transmitted infections, emergency contraception, pre-exposure prophylaxis and post-exposure prophylaxis for HIV are currently available in Malawi and many countries [24]. Due to stigma and discrimination and non-responsive health care providers, FSWs will not easily access those helpful health care services. Female sex workers should be in good health, they should not be tricked and their clients should not take advantage of their poor socio-economic status to dictate the way sexual intercourse must be done. Through reproductive health services integrated in HIV and AIDS programs, and tailored to the nature of sex work, FSWs can overcome most of their reproductive health challenges. There is a need to remove all barriers hindering FSWs access to health care services.

**Table 2:** The correlates of pregnancy among female sex workers (FSWs) in semi urban Blantyre, Malawi

Characteristics	Pregnancy		COR (95%CI)	*p-*Value	AOR (95%CI)	*p-*Value
	Yes	No				
**Place of birth**						
Urban	37 (40.22)	55 (59.78)	0.496 (0.281–0.875)	0.016	0.308 (0.137–0.690)	0.004
Rural	61 (57.55)	45 (42.45)				
**Inconsistence condom use**						
No	43 (35.83)	77 (64.17)	4.282 (2.319–7.906)	0.000	5.259 (2.289–12.081)	0.000
Yes	55 (70.51)	23 (29.49)				
**To be respected as a mother**						
No	17 (21.25)	63 (78.75)	8.112 (4.184–15.727	0.000	11.844 (4.566–30.720)	0.000
Yes	81 (68.64)	37 (31.36)				
**Request from a steady partner**						
No	29 (27.62)	76 (72.38)	7.534 (4.006–14.167	0.000	5.071 (2.145–11.990)	0.000
Yes	69 (74.19)	24 (25.81)				
**Missing contraceptive pills**						
No	33 (38.82)	52 (38.82)	2.101 (1.182–3.732)	0.011	3.490 (1.299–9.370)	0.013
Yes	64 (57.14)	48 (42.86)				

*AOR* Adjusted Odds Ratio, *COR* Crude Odds Ratio, *CI* Confidence Interval

Study findings suggest hat in Mombasa, Kenya 80% of FSWs reported inconsistent condom use with their steady partners [25]. In our study almost half (49%) of the study participants have steady partners. Our research also unveiled that steady partners’ request of having children with FSWs is highly associated with pregnancy among FSWs. Female sex workers and their steady clients face dilemma on the use of condoms during sexual intercourse [26]. Given the close ties between HIV, STI and pregnancy and high prevalence of HIV in FSWs community there is a critical need for accessible and targeted PMTCT services, combined with other reproductive health services such as family planning, antenatal, postnatal care and rearing children [2, 16].

Female sex workers’ experiences of pregnancies differ from other women of reproductive age because they have a broader range of partners. They may have many steady partners, casual or anonymous partners [2, 27]. According to our study finding some circumstances are also similar. Female sex workers who reported that they want to be respected as mothers had high odds of pregnancy comparing to the ones who did not have the same desire (Table 2). Study findings in several countries show that with childbearing FSWs and other women of the same reproductive age earn respect [2, 11]. Children also solidify relationships between women and their partners. Our findings concur with those previous study’ findings but many other studies suggest that pregnancy has a negative impact on the ability of sex trade due to the additional stigma of being a pregnant sex worker, or being perceived as less sexually attractive [28]. Contrary to the previous study findings, [2, 11] we did not find the relationship between FSWs’ pregnancy and the following variables: mothers’ obligations, rape, challenges in accessing contraceptives, extension of the clans.

### Limitations of the study

Sex work is a sensitive issue; as such there is potential for social desirability bias which in turn underestimates the magnitude of studied issue in FSWs community. Since great care was taken to assure the study participants of confidentiality of the information and privacy of the research participants, we hope that this problem was minimized. We recruited the first FSWs as seeds to assist in snowballing sampling of study participants. This method can have a potential sampling bias because people refer those whom they know and/or have similar traits. Therefore our findings cannot be generalized to the complete FSWs population. The research was cross sectional, it is not possible to assign causation between the study outcome and the variables. There is also a possibility of recall bias and misreporting of personal experiences.

## Conclusion

In this study we have many young FSWs (45.5%) as compared to the remaining age groups. Due to inconsistent condom use in this group, there is high likelihood of teenage pregnancy. The later is associated with sexually transmitted infections (STI) infections including HIV, life threatening maternal and neonatal conditions such preeclampsia and premature birth, unsafe abortions and post- abortion infections [29, 30]. In addition teenage and pregnant female sex workers living with HIV will have clinical and psychosocial concerns which require proper attention by health care providers [29, 30]. The maternal and neonatal complications can be prevented if these pregnancies are avoided or delayed. There is a need for access to reproductive health services integrated in antiretroviral therapy (ART) programs.

Our study findings suggest that FSWs experience pregnancy under diverse circumstances. Inconsistent condom use with the clients, desire to be respected as mothers, request from a steady partner, and missing contraceptive pills are associated with pregnancy in FSWs community in semi urban Blantyre. All these correlates have many risks because there is an obvious unprotected heterosexual intercourse in the process. For the safety of both partners and their unborn babies, intervention such as pre-and post-exposure prophylaxis for HIV, prevention of mother to child transmission (PMTCT) can be used to improve these pathways. The health promotion programs should be tailored with FSWs needs as well as the complex environment they operate from. It is important to recognize the child bearing desires and circumstances of FSWs in order to inform health programs responsive to their needs. Qualitative studies should evaluate men drivers to have children with FSWs.

## Data Availability

The data sets used and/or analyzed during this study are available from the corresponding author on reasonable request.

## References

[1] Weldegebreal R, Melaku YA, Alemayehu M, Gebrehiwot TG (2015). Unintended pregnancy among female sex workers in Mekelle city, northern Ethiopia: a cross-sectional study. BMC Public Health.

[2] Beckham SW, Shembilu CR, Brahmbhatt H, Winch PJ, Beyrer C, Kerrigan DL (2015). Female sex workers’ experiences with intended pregnancy and antenatal care services in southern Tanzania. Stud Fam Plan.

[3] Erickson M, Goldenberg SM, Ajok M, Muldoon KA, Muzaaya G, Shannon K (2015). Structural determinants of dual contraceptive use among female sex workers in Gulu, northern Uganda. Int J Gynaecol Obstet.

[4] Southrland EG, Alaii J, Tsui S, Luchters S, Okal J, King’ola N, Temmerman M, Janowitz B (2011). Contraceptive needs of female sex workers in Kenya – across-sectional study. Eur J Contraception Reprod Health Care.

[5] Luchters S, Chersich MF, Jao I, Temmerman M, Schroth A, Chidagaya S, Mandaliya K (2007). Acceptability of the diaphragm in Mombasa Kenya: a 6-month prospective study. Eur J Contracept Reprod Health Care.

[6] Thomsen SC, Ombidi W, Toroitich-Ruto C, Wong EL, Tucker HO, Homan R, Kingola N, Luchters S (2006). A prospective study assessing the effects of introducing the female condom in a sex worker population in Mombasa, Kenya. Sex Transm Infect.

[7] Basu A, Dutta MJ (2011). 'We are mothers first': Localocentric articulation of sex worker identity as a key in HIV/AIDS communication. Women Health.

[8] Schwartz S, Papworth E, Thiam-Niangoin M, Abo K, Drame F, Diouf D, Bamba A, Ezouatchi R, Tety J, Grover E, Baral S (2015). An urgent need for integration of family planning services into HIV care: the high burden of unplanned pregnancy, termination of pregnancy, and limited contraception use among female sex workers in Cote d’Ivoire. J Acquire Immune Defic Syndr.

[9] Decker MR, HL MCC, Phenengsamlan D, Janyam S, GRS I, Silverman JG (2010). Violence, victimization, sexual risk and STI symptoms among a natural sample of FSWs in Thailand. Sex Transm Infect.

[10] Yam EA, Kidann A, Burnett-Zieman B, Pilgrim N, Okal J, Bakele A, Gudeta D, Caswell G (2017). Pregnancy experiences of female sex Workers in Adama city City, Ethiopia: complexity of partner relationships and pregnancy intentions. Stud Fam Plan.

[11] Beckham SW, Shembilu CR, Winch PJ, Beyrer C, Kerrigan DL (2015). ‘If you have children, you have responsibilities’: motherhood, sex work and HIV in southern Tanzania. Cult Health Sex.

[12] Chersich MF, Luchters S, Ntaganira I, Gerbase A, Lo YR, Scorgie F (2013). Priority interventions to reduce HIV transmission in sex work settings in sub-Saharan Africa and delivery of these services. J Int AIDS Soc.

[13] Papworth E, Schwartz S, Ky-zerbo O, Leistman B, Ouedraogo G, Samadoulougou C, Grosso A, Drame F, Diouf D, Ketende SC (2015). Mothers who sell sex: a potential paradigm for integrated HIV, sexual, and reproductive health interventions among women at high risk of HIV in Burkina Faso. J Acquir Immune Defic Syndr.

[14] Duff P, Shoveller J, Jill C, Feng C, Rachel N, Shannon K (2015). Sex work and motherhood: social and structural barriers to health and social services for pregnant and parenting street and off-street sex workers. Health Care Women Int.

[15] Deering KN, Shannon K, Sinclair H, Parsad D, Gilbert E, Tyndall MW (2009). Piloting a Peer-Driven Intervention Model to Increase Access and Adherence to Antiretroviral Therapy Women in Vancouver. AIDS Patient Care STDS.

[16] Pauw I, Brener L (2003). ‘You are just whores—you Can’t be raped’: barriers to safer sex practices among women street sex Workers in Cape Town. Cult Health Sex.

[17] Murray L, Moreno L, Rosario S, Ellen J, Sweat M, Kerrigan D (2006). The role of relationship intimacy in consistent condom use among female sex workers and their regular paying partners in the Dominican Republic. AIDS Behav.

[18] Todd CS, Nasir A, Stanekzai MR, Scott PT, Close NC, Botros BA, Strathdee SA, Tjaden J (2011). HIV awareness and condom use among female sex workers in Afghanistan: implications for intervention. AIDS Care.

[19] Tran TN, Detels R, Lan HP (2006). Condom use and its correlates among female sex workers in Hanoi, Vietnam. AIDS Behav.

[20] Johnston CL, Callon C, Li K, Wood E, Kerr T (2010). Offer of financial incentives for unprotected sex in the context of sex work. Drug Alcohol Rev.

[21] Bucardo J, Semple SJ, Fraga-Vallejo M, Davila W, Patterson TL (2004). A qualitative exploration of female sex work in Tijuana,Mexico. Arch Sex Behav.

[22] Strathdee SA, Philbin MM, Semple SJ, Lozarda R, Orozovich P, Pu M, Staines-Orozco H, Fraga-Vallejo M, Amaro H, Delatorre A, Magis-Rodriguez C, Patterson TL (2008). Characteristics of female sex workers with US clients in two Mexico-US border cities. Sex Transm Dis.

[23] Mukumbang FC (2017). Actions of female sex workers who experience male condom failure during penetrative sexual encounters with clients in Cape Town: implications for HIV prevention strategies. S Afr J HIV Med.

[24] Ministry of Health and Population, Malawi (2018). Clinical Management of HIV In Children and Adults. 2018.

[25] Luchters S, Chersich MF, Rinyiru A, Barasa M-S, King’ola N, Mandaliya K, Bosire W, Wambugu S, Mwarogo P, Temmerman M (2008). Impact of five years peer-mediated interventions on sexual behavior and sexual transmitted infections among female sex workers in Mombasa, Kenya. BMC Public Health.

[26] Okal J, Stadler J, Ombidi W, Jao I, Luchters S, Temmerman M, Chersich MF (2008). Secrecy, disclosure and accidental discovery: perspectives of diaphragm users in Mombasa, Kenya. Cult Health Sex.

[27] Duff P, Shoveller J, Feng C, Ogilvie G, Montaner J, Shannon K (2015). Pregnancy intentions among female sex workers: recognizing their rights and wants as mothers. J Fam Plan Reprod Healthc.

[28] Luchters S, Bosire W, Feng A, Richter ML, King’ola N, Ampt F, Temmerman M, Matthew F, Chersich MF (2016). ‘A Baby Was an Added Burden’: Predictors and Consequences of Unintended Pregnancies for Female Sex Workers in Mombasa, Kenya: A Mixed-Methods Study. PLoS ONE.

[29] Yakubu I, Salisu WJ (2018). Determinants of adolescent pregnancy in sub-Saharan Africa: a systematic review. Reprod Health.

[30] Christofides NJ, Jewkes RK, Dunkle KL, Nduna M, Shai NJ, Sterk C (2014). Early adolescent pregnancy increases risk of incident HIV infection in the eastern cape, South Africa: a longitudinal study. J Int AIDS Soc.

